# Clinical Mentorship of Nurse Initiated Antiretroviral Therapy in Khayelitsha, South Africa: A Quality of Care Assessment

**DOI:** 10.1371/journal.pone.0098389

**Published:** 2014-06-02

**Authors:** Ann Green, Virginia de Azevedo, Gabriela Patten, Mary-Ann Davies, Mary Ibeto, Vivian Cox

**Affiliations:** 1 School of Public Health and Family Medicine, University of Cape Town, Cape Town, South Africa; 2 Department of Health, City of Cape Town, Cape Town, South Africa; 3 Médecins Sans Frontières Khayelitsha, Khayelitsha, South Africa; Fundacion Huesped, Argentina

## Abstract

**Introduction:**

To combat the AIDS epidemic and increase HIV treatment access, the South African government implemented a nurse-based, doctor-supported model of care that decentralizes administration of antiretroviral treatment (ART) for HIV positive patients through nurse initiated and managed ART. Médecins Sans Frontières (MSF) implemented a mentorship programme to ensure successful task-shifting, subsequently assessing the quality of clinical care provided by nurses.

**Methods:**

A before-after cross-sectional study was conducted on nurses completing the mentorship programme in Khayelitsha, South Africa, from February 2011-September 2012. Routine clinical data from 229 patient folders and 21 self-assessment questionnaires was collected to determine the number of patients initiated on ART by nurses; quality of ART management before-after mentorship; patient characteristics for doctor and nurse ART initiations; and nurse self-assessments after mentorship.

**Results:**

Twenty one nurses were authorized by one nurse mentor with one part-time medical officer's support, resulting in nurses initiating 77% of ART eligible patients. Improvements in ART management were found for drawing required bloods (91% vs 99%, p = 0.03), assessing adherence (50% vs 78%, p<0.001) and WHO staging (63% vs 91%, p<0.001). Nurse ART initiation indicators were successfully completed at 95–100% for 11 of 16 indicators: clinical presentation; patient weight; baseline blood work (CD4, creatinine, haemoglobin); STI screening; WHO stage, correlating medical history; medications prescribed appropriately; ART start date; and documented return date. Doctors initiated more patients with TB/HIV co-infection and WHO Stage 3 and 4 disease than nurses. Nurse confidence improved for managing HIV-infected children and pregnant women, blood result interpretation and long-term side effects.

**Conclusions:**

Implementation of a clinical mentorship programme in Khayelitsha led to nurse initiation of a majority of eligible patients, enabling medical officers to manage complex cases. As mentorship can increase clinical confidence and enhance professional development, it should be considered essential for universal ART access in resource limited settings.

## Introduction

South Africa is one of the countries most severely affected by the AIDS pandemic, with an estimated 5.6 million people living with HIV & AIDS (PLWHA) and the largest antiretroviral treatment programme in the world [1]. Addressing the large numbers of patients requiring ART initiation and management has become a key priority. To increase the number of people that can access HIV care and management, the South African government implemented a task-shifting model in 2011 that certifies nurses for the administration of antiretroviral treatment (ART) for HIV positive patients [2]. Under this model of care, trained nurses initiate patients onto ART and continue management of their subsequent treatment. However, limitations to human resource capacity, infrastructure, drug supplies, and a lack of clinical mentorship for newly trained nurses have all contributed to inefficiencies in the system.

### Task Shifting for ART Management and ART Initiation

As in South Africa, human resource constraints within a healthcare system often lead to some form of task-shifting, or re-distribution of health-related tasks to a cadre of lower level professionals [3]. A systematic review conducted in 2010 on task shifting for HIV care and treatment in Africa found that task shifting is an effective strategy for combating shortages in human resources for health in HIV care and treatment [4]. Specifically, the *Comprehensive International Programme of Research on AIDS* (CIPRA-SA) and *Streamlining Tasks and Roles to Expand Treatment and Care for HIV* (STRETCH) randomized control trials conducted in South Africa found nurse monitored ART to be non-inferior to doctor monitored therapy [5] [6] [7]. Some doctor initiated patients, down-referred to a primary care facility managed by nurses, had twelve-month outcomes for stable ART patients that were as good as and, in some cases, better than those patients that were maintained under hospital-based ART care [8]. The time saving benefits of nurse initiated and managed ART (NIMART) [9] and proven high levels of nurse compliance with national treatment guidelines [10], support the notion that improved access to life-saving ART treatment in resource limited settings can be realized through shifting of tasks to medical assistants and nurses [11]. In fact, in 2008, the WHO released a new set of guidelines and recommendations on task shifting that assigned tasks related to ART initiation and management under the purview of nurses as well as doctors [3]. Despite this, there is still debate over whether nurse initiation of ART is sustainable and whether there are negative impacts on quality of care [12], [13], [14].

Most data on NIMART comes from trials rather than routine care settings, and, outside of the STRETCH trial, there is little research that explores the performance of NIMART graduates in the clinical management and initiation of ART and the quality of care provided. According to a recent study of initiation rates for NIMART graduates within seven South African provinces, of the 126 trained nurses that were sampled, only 72 (62%) nurses proceeded to actually initiating patients onto ART [15]. The South African Department of Health has since set a goal to have nurses initiating 85% of patients eligible for ART by 2016 [16]. Under this new model of care, clinical mentorship is a key factor in the process for NIMART graduates to independently initiate ART; however, there is little data on the effectiveness of clinical mentorship programs [17]. The objective of this study is to explore the quality of clinical care received by patients initiated or managed by nurses completing a clinical mentorship programme led by Médecins Sans Frontières (MSF) in Khayelitsha, South Africa.

The study had two main aims: to describe the characteristics of ART initiations done by nurse graduates of the MSF NIMART mentorship programme; to measure the quality of nurses' initiation and long-term clinical management of patients continuing ART in Khayelitsha City Health primary health care clinics.

## Methods

### Ethics Statement

Ethics approval for this study was provided by the University of Cape Town Human Research Ethics Committee, with permission to conduct the research also obtained from the Western Cape Provincial Research Committee, as well as the sub-district supervisor and facility manager for each selected clinic.

The basic principles of the South African Medical Research Council were upheld, which include autonomy, beneficence, non-maleficence and justice. Routine sources of data were used from each clinic, thus consent was not obtained from the individual patients or the nurse participants. The use of patient and nurse data without consent was approved by the ethics committees that approved this study, as both patient and nurse data was kept anonymous. The data collected is similar to the data reviewed during routine patient folder checks for quality control, and the nurses were aware of others accessing this information regarding their performance.

### Study Overview

This was a before-after cross-sectional study involving patient folder reviews of clinic adult ART stationery and self-assessments completed by NIMART nurses before and after mentorship was undertaken. The study was conducted in the South African township of Khayelitsha, a sub-district with an estimated population of approximately 500,000 inhabitants, located on the outskirts of Cape Town in Western Cape Province, South Africa [18]. The healthcare needs of township residents are served through 8 primary health clinics, three community health centres and a recently developed district hospital. As of 2010, Khayelitsha's HIV burden is one of the largest in the country, with an antenatal HIV prevalence of 26% [19]. The high volume of patients requiring ART initiation and management highlights the need for task shifting in order to ensure access to care for all eligible patients. Following the South African government's 2009 mandate for universal ART access, the programme expanded rapidly with all 11 clinics providing access to ART by 2011.

**Figure 1:**
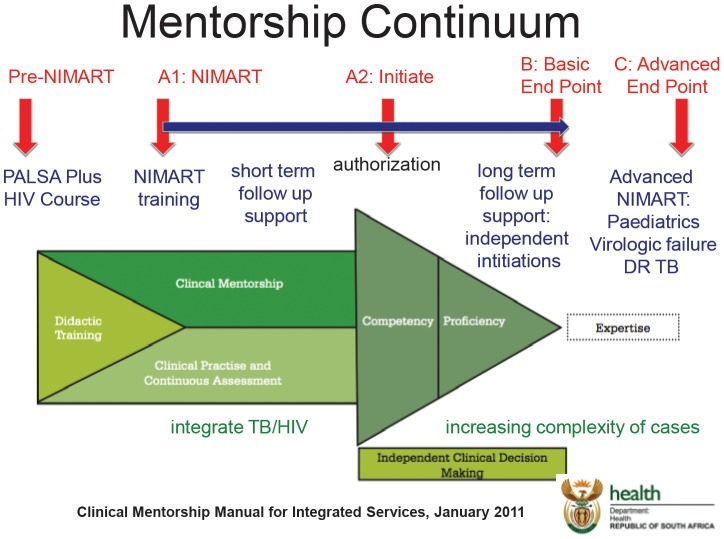
NIMART Clinical Mentorship Model for Integrated Services. Pre-NIMART: *PALSA Plus HIV course* training on use South Africa's approved algorithmic tool for diagnosis and treatment of common opportunistic infections; A1: *NIMART training* based on South African Ministry of Health guidelines; A2: *Authorization to initiate* is granted based on three required criteria: completion of basic HIV course, PALSA Plus HIV training, and 40 hours of one-on-one mentorship; B: *Basic End Point* is reached whereby the authorized nurses is conducting independent initiations; C: *Advanced End Point* is reached when skill level is expanded to include management of paediatric patients, virologic failure, and drug-resistant TB.

Since February 2011, MSF has been implementing a NIMART training and mentorship program for nurses in Khayelitsha to become accredited to initiate ART [20]. As of December 31, 2013, 81 nurses have been or are being mentored through the MSF NIMART programme; 70 of which have been authorised to initiate ART independently. The mentorship programme follows the nurse-based, doctor-supported ART service model promoted by the Western Cape's Provincial HIV & AIDS, STI, and TB Directorate as depicted in Figure 1 [21]. The pre-requisites for NIMART authorization in the Western Cape of South Africa are: 1. Completion of a basic HIV course (from a list of approved courses that is adjusted as new courses become available); 2. Completion of PALSA Plus HIV training (a training on how to use South Africa's approved algorithmic tool for diagnosis and treatment of common opportunistic infections); 3. 40 hours of one-on-one mentorship.

This study focused on nurses completing the MSF NIMART mentorship programme and working in eight Khayelitsha facilities; a random sample of these nurses was chosen, with at least one nurse from each facility. Data from programme records was used to determine the number of nurses completing the NIMART mentorship program and receiving authorization to prescribe ART as well as the number and proportion of all patient ART initiations done by NIMART mentored nurses. Data was also collected through patient folder review to: 1) compare the quality of nurse ART management before and after NIMART training; 2) describe the quality of nurse ART initiations; and 3) compare patient characteristics for doctor and nurse ART initiations. Quality of ART initiation and management was assessed based on correct completion of standard clinical stationery components in use across all the study clinics. Data on patient characteristics included age, gender, weight, body mass index (BMI), creatinine and creatinine clearance, haemoglobin, alanine aminotransferase (ALT), tuberculosis (TB) status, pregnancy status, and WHO stage. Additionally, qualitative data comparing the level of nurse confidence before and after NIMART training was collected from a self-assessment survey that captured each nurse's perception of individual clinical confidence for a list of competencies on a level of 1-5 (1 =  not very confident; 2 =  somewhat confident; 3 =  confident; 4 =  very confident; 5 =  confident if using manual).

The study period for reporting the number and proportion of NIMART nurse patient initiations and the number of nurses completing the NIMART mentorship programme spans from February 2011 to September 2012. The period for measuring quality of nurse ART management prior to mentorship was determined based on the nurse's authorization date, using data from February 2011 through the start date of each nurse's participation in the NIMART program, the “before period”. The period for assessing ART management after mentorship was defined as the post-authorization date to September 2012, the “after period”. For measuring the quality of nurse initiations and for collecting patient characteristics of nurse and doctor ART initiations, the study period was based on nurse authorization date through September 2012. The self-assessment questionnaires were completed on the start date of the NIMART mentorship programme. The same questionnaire was then repeated on the final date of the respective training.

**Figure 2:**
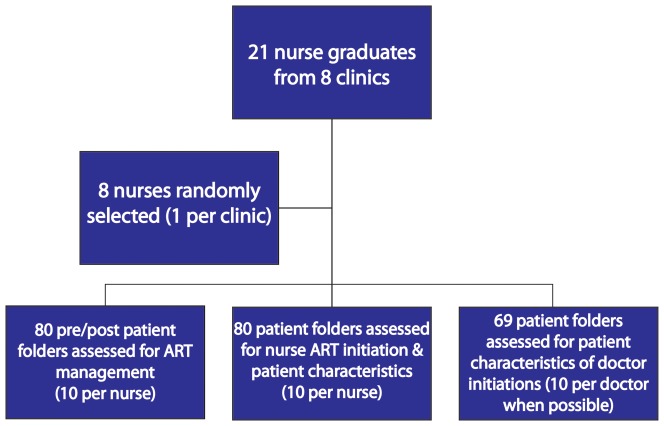
Sample selection flow diagram.

For data on quality of nurse ART management, a nurse graduate was randomly selected from a list of each of the study clinics' mentored nurses. Patient folders were randomly sampled for the selected nurses, identifying ten folders for the before period and ten folders for the after period. Eighty patient folders in total were reviewed for the before and after periods, assessing quality of nurse ART management. An additional 80 folders were selected for assessing quality of nurse graduate ART initiation. The clinical stationery components chosen to assess quality of ART management and initiation included variables related to necessary laboratory testing, patient counselling, medication and side effect management, and adherence. To compare patient characteristics with those initiated on treatment by doctors, folders were randomly selected from a sampling frame of all doctor-led ART initiations at each clinic during the study period. All random selection was done using a random number generator programme (random.org) that includes a list randomizer function [22]. Figure 2 depicts a flow diagram for the sample selection process.

Programme data was summarized using appropriate descriptive statistics of frequency and percentage for categorical variables. The quality of nurse ART management and initiations were analysed by calculating the proportion of the chosen clinical stationery components correctly completed within the 10 folders reviewed for each nurse graduate and conducting chi-squared tests. Patient characteristics of both doctor and nurse ART initiations were analysed by calculating medians and inter-quartile ranges for continuous data and proportions for categorical data. Chi-squared tests were calculated where appropriate. Self-assessment data was summarized using descriptive statistics and rank sum tests. Where applicable the data was analysed using STATA statistical software version 12 [23].

## Results

There were 21 nurse graduates from the NIMART mentorship program during the study period. In the month of September 2012, 77% (173/223) of ART initiations in study clinics were done by nurse graduates. Three graduates resigned from their position during the study period. Twenty-one self-assessments were conducted before the NIMART mentorship program and again after completion of the program, two for each nurse graduate. Two nurses' before and after surveys for self-assessment analysis were excluded due to differing survey formats in the before and after period.

In terms of assessing the quality of nurse ART management following the NIMART mentorship program, there were significant improvements in the collection of required blood tests, patient HIV staging and nurse adherence assessment and documentation(Table 1). Of the 80 patient folders reviewed in each study period, one patient was referred to a doctor in the before period and two patients in the after period.

**Table 1:** Quality of Nurse ART Management in Khayelitsha Clinics n = 80 patient clinical folders reviewed.

Indicator	Before	%	After	%	p-value
ART date recorded	80	100	79	98.8	0.316
Last viral load>400	4/54*	7.41	3/65*	4.6	0.519
Treatment plan documented for patients with viral load >400	2/4*	50	1/3*	33.3	0.659
Creatinine clearance recorded	13/74*	17.56	10/65*	15.4	0.73
Documented result of most recent bloods	74	92.5	75	93.8	0.755
Last required bloods drawn	73	91.25	79	98.8	**0.03**
TB screen done	63/76*	82.89	68/74*	91.9	0.098
STI screen done	71	88.75	75	93.8	0.263
Family planning offered	33/56*	58.93	37/55*	67.3	0.362
Pap smear recorded /referenced	4/60*	6.67	6/57*	10.5	0.455
Clinical presentation entered	80	100	80	100	
Problem list entered	33	41.25	36	45	0.632
Adherence assessed and documented	40	50	62	77.5	**<0.001**
Stage entered	50	62.5	73	91.3	**<0.001**
Stage correlates with known medical history	45	88	58	79.5	0.119
Treatment plan documented	58	72.5	66	82.5	0.13
Referred to doctor	1	1.25	2	2.5	0.56
Medication entered	80	100	80	100	
ARV dose entered correctly	64	80	64	80	
Cotrimoxazole prescribed	12	15	8	10	0.339
Should Cotrimoxazole have been prescribed	21	26.25	15	18.8	0.256

*Number of patients where particular indicator is applicable.

Analysis of nurse-managed ART initiation revealed that nurse graduates documented baseline CD4 count, calculated and entered patient HIV stage in accordance with patient medical history, and documented the ART initiation date and the prescribed medication 100% of the time(Table 2). In more than 90% of records reviewed, nurse graduates documented patient weight, past medical history, prior ART history and haemoglobin; screened for sexually transmitted infections; and documented a treatment plan and a return visit date. Pap smear data was only recorded in 14% of female records reviewed. Cotrimoxazole was prescribed in 76% of the patients requiring it.

**Table 2:** Quality of Nurse ART Initiations in Khayelitsha Clinics n = 80.

	Number recorded	%
Patient weight documented	76	**95**
Patient BMI documented	60	75
Past medical history documented	73	91.3
Prior ART/history documented	73	91.3
If yes, was patient discussed with doctor	0	
Documented as ‘No ART history’	33	41.3
Baseline CD4 documented	80	**100**
Creatinine documented	79	**98.8**
Creatinine clearance (CrCl) documented	43	53.8
IfCrCl<50, was patient discussed with doctor	0	
Not applicable	77	41.3
IfCrCl<50 and discussed by doctor, treatment documented	0	
Haemoglobin documented	78	**97.5**
Alanine aminotransferase (ALT) documented	70	87.5
IfALT>100, was patient referred to doctor	0	
ART initiation date recorded	80	**100**
TB screen done	61	76.3
On treatment	17	21.3
Investigation pending	1	1.3
STI screen done	76	**95**
Eligible for family planning	59	73.8
Family planning offered	42	71.2
Eligible for pap smear	59	73.8
Pap smear date recorded or referenced	8	13.6
Clinical presentation entered	80	**100**
Problem list entered	62	77.5
Stage entered	80	**100**
Stage correlates with known medical history	80	**100**
Treatment plan entered	74	92.5
Was patient seen or discussed with doctor in prior month	19	23.8
Medication entered	80	**100**
ARV dose entered correctly	66	82.5
Should Cotrimoxazole have been prescribed	41	51.3
Cotrimoxazole prescribed	30	37.5
Return date documented	79	**98.8**

Patients initiated on treatment by doctors had more advanced disease compared with those initiated by nurses(Table 3); median weight, BMI and haemoglobin levels were lower, and the median ALT was higher. A greater proportion of doctor-initiated patients had active TB and WHO Stage 3 or 4 disease.

**Table 3:** Patient Characteristics n = 80 n = 69.

	Nurse	Doctor	p-value
Age (median)	28 (IQR 24–35)	31 (IQR 24–39)	0.5654
Gender			0.352
Male	20 (25%)	22 (28%)	
Female	60 (75%)	47 (62%)	
Weight (median)	66 (IQR 61–78)	59 (IQR 52–70)	**<0.001**
Weight not recorded	3	0	
Infants	0	2	
BMI (median)	26 (IQR 23–31)	22 (IQR 19–26)	**0.0014**
Weight not recorded	3	0	
BMI not recorded	19	25	
Infants	0	2	
Creatinine (median)	62 (IQR 50–73)	66 (IQR 51–81)	0.2161
missing	1	2	
Creatinine clearance (median)	103 (IQR 80.5–125.5)	120 (IQR 71.5–154)	0.5926
missing	38	41	
Haemoglobin (median)	12 (IQR 11–13)	11 (IQR 9–13)	0.0463
missing	2	8	
Alanine aminotransferase (median)	18 (IQR 14–24)	26 (IQR 19–38)	**<0.001**
missing	11	6	
TB status			**<0.001**
On TB treatment	17	27	
Screen positive	0	0	
Screened negative	61	30	
Investigation Pending	1	1	
Not Screened	1	11	
Pregnant	3	1	0.453
WHO stage			**<0.001**
Stage 1	42	11	
Stage 2	11	13	
Stage 3	22	28	
Stage 4	5	12	
Missing Stage	0	5	

Self-assessment data revealed a significant increase in nurse confidence level for nearly all clinical tasks measured, notably for management of children, body mass index calculation, creatinine clearance, prescribing with concurrent illness, recognition and management of side effects, pregnancy planning in HIV positive patients, and non-drug management and administration of post-exposure prophylaxis(Table 4).

**Table 4:** Nurse Graduate Self-assessment n = 21 n = 21.

	Pre	Post	
	mean score	mean score	p-value
**Section 1: Baseline Knowledge**			
Modes of transmission - adults	3.3	3.8	0.0083
Modes of transmission - children	2.905	3.737	0.0060
Clinical course - adults	2.619	3.7	0.0016
Clinical course - children	2.278	3.5	0.0004
Signs and symptoms - adults	3.19	3.905	0.0014
Signs and symptoms - children	2.6	3.65	0.0043
Use of manuals –positive prevention& HIV	3.25	3.75	0.0247
Use of Department of Health stationery	2.8	3.789	0.0003
Core differences between adults & children	2.263	3.524	0.0013
**Section 2: Before ARVs**			
HIV counselling and testing - adults	3.25	3.85	0.0008
HIV counselling and testing - children	2.111	3.714	<0.0001
Diagnosis - adults	3	3.8	0.0003
Diagnosis - children	2	3.55	<0.0001
Staging - adults	3.3	3.905	0.0053
Staging - children	1.8	3.619	<0.0001
Body Mass Index calculation and management	3.2	3.952	<0.0001
Paediatric weight chart and management	1.789	3.474	<0.0001
TB screening - adults	3.476	3.857	0.1279
TB screening - children	2.476	3.476	0.0046
Pap smears and HIV	2.95	3.8	0.0007
Common opportunistic infections - adults	2.952	3.762	0.0013
Common opportunistic infections - children	2.1	3.381	0.0003
TB diagnosis and management - adults	3.05	3.571	0.0799
Drug side effects	2.905	3.824	0.0187
Cotrimoxazole use - adults	3.429	3.714	0.1654
Cotrimoxazole use - children	2.048	3.81	<0.0001
**Section 3: Starting ARVs**			
Eligibility criteria to start - adults	2.857	3.667	0.0029
Eligibility criteria to start - children	1.65	3.429	<0.0001
Fast-tracking criteria	2.762	3.75	0.0001
Blood result interpretation - adults	2.5	3.762	0.0003
Working withcreatinine clearance	2.1	3.905	<0.0001
Individual ART contra-indications	2.35	3.81	0.0004
Pre-start adherence counseling	2.55	3.7	0.0003
Department of Health1st line regimen - adults (ARVs, doses, side effects)	3	3.714	0.0870
Department of Health1st line regimen – children (ARVs, doses, side effects)	1.65	3.476	<0.0001
Prescribing w concurrent illnesses - TB, epilepsy, hypertension, diabetes	1.857	3.75	<0.0001
Normal course on ARVs	2.737	3.905	<0.0001
Side effects and their management:			
Skin, haematology, liver, kidney, psychiatric	2.143	3.6	0.0010
Recognition of side effects	2.667	3.429	0.0530
Understanding IRIS	2.35	3.571	0.0031
Long-term side effects	2.053	3.667	0.0002
Failing regimen - warning signs	2.4	3.667	0.0002
Diagnosis - clinical, immunological, virological	1.842	3.619	<0.0001
When to refer	2.333	3.857	<0.0001
**Section 4: HIV & Pregnancy**			
Family planning	3.095	3.762	0.0012
Wanting to fall pregnant	2.19	3.9	<0.0001
During pregnancy	2.571	3.7144	0.0005
After pregnancy - mother	2.35	3.762	0.0003
After pregnancy - child	2.1	3.5	0.0006
**Section 5: Post-exposure Prophylaxis**			
Non-drug management and administration	2.05	3.762	<0.0001
Drug regimen and indications	2.15	3.65	0.0006
**Section 6: General**			
Management of weight loss	2.7	3.571	0.0066
Routine clinical examination	2.438	3.667	0.0001
Cardiac failure	1.667	3.118	0.0005
Enlarged liver	1.81	3.313	0.0009
Neck stiffness	2.15	3.5	0.0009
Procedures for lactate testing	1.571	3.2	0.0001
Understanding the register	2.111	3.167	0.0186

Following the mentorship program nurses reported the lowest confidence scores in the following areas: cardiac failure, understanding of the ARV register, and procedures for lactate testing. Confidence did not significantly increase after mentoring in the following areas: TB screening, diagnosis and management in adults; cotrimoxazole prescription in adults; and management of the Department of Health first line regimen in adults. However, the confidence rates for these areas were moderately high (3–3.5) in the pre-test.

## Discussion

This study found that a NIMART mentorship program successfully resulted in a majority of ART initiations conducted by nurses, which is an encouraging step toward reaching the South African National Strategic Plan's goal of 85% of eligible patients being initiated by nurses by 2016. The quality of ART management provided by nurses improved, as well as the nurses' confidence in their knowledge and management of HIV. The quality of nurse initiation of patients onto ART following mentorship was satisfactory, allowing doctors to focus on patients with more advanced disease.

The study findings complement previous NIMART studies conducted in sub-Saharan Africa, whereby nurse ART management and initiation are recommended as effective strategies for addressing HIV care and treatment in low-resource settings. [4] However, this study has shown a higher percentage of nurse initiations than other studies. [15]

The MSF NIMART programme was conducted in line with the South African Ministry of Health (MoH) guidelines on NIMART mentorship with the intent to eventually hand over the programme. For this reason the results of the programme are seen as sustainable. MSF also supported the development of a Western Cape Provincial Trainer-of-NIMART Mentors course in 2011, and this course is now managed completely by the MoH without MSF involvement, producing +/− 20 new mentors a year.

### Quality of Nurse ART Management

The nurse graduates successfully completed a number of ART management indicators both before and after the mentorship program, indicative of appropriate care of those on ART. The consistency in documentation is perhaps due to the fact that the nurses participating in the mentorship programme had significant prior experience in managing ART. Despite this, there are still some areas of concern in nurse management of ART, such as documentation of pap-smears for female patients. Consistent with other study findings [24], this is a topic to be included in future nurse mentorship programme content, especially given the high levels of cervical cancer throughout South Africa [25]. The absence of documented TB screening for all patients in an area with a high burden of TB-disease is also disconcerting, although improvements were observed after mentorship. Another concern is the low calculation rates of creatinine clearance (CrCl) for follow-up monitoring, although this may be attributed to the ART stationery in use not having a specific field to document the result. The ratio of Cotrimoxazole (Bactrim) being prescribed in patients that were eligible for it was also low. As this is an effective, affordable method for strengthening the immune system of HIV infected patients, it is imperative that the mentorship program place a strong focus on improving these rates moving forward.

Our results show that few patients were referred to doctors within the study clinics. This may result from the fact that the patients seen by nurses were characteristically not as ill, rather than nurse graduates not appropriately seeking professional guidance from doctors. The low level of doctors present within these clinics may contribute to the low levels of referrals. It is also possible that nurses consult a doctor or nurse for advice during the initiation process without documenting this advice in the patient folder.

Following completion of the NIMART mentorship program, there were improvements in indicators for drawing required bloods, assessing adherence and WHO staging. While the significant improvement in assessing adherence is encouraging (77.5%), there is still substantial room for improvement. Monitoring of patient adherence is crucial considering it is a key barrier to successfully keeping patients on treatment, and it will be important to continue focus on this area in future mentorship trainings.

### Quality of Nurse ART Initiation

Overall, the high levels of completion for the majority of clinical indicators for ART initiation among nurse graduates suggest that such task shifting has been a success. While creatinine levels were consistently recorded at ART initiation, the calculation of creatinine clearance (CrCl) was often not included in the patient folder. Nurse graduates seem to pay more attention to creatinine clearance (CrCl) when initiating patients onto ART compared to when managing ART in patients already initiated. This may be due to a deeper sense of responsibility felt when personally initiating patients. Similar to nurse ART management results, further improvements are needed in rates of pap smears, prescription of Cotrimoxazole, and TB screening. There are low numbers of TB co-infected patients among those seen by nurse graduates, possibly due to the level of TB service integration at the study clinics.

### Patient Characteristics between Doctor & Nurse Initiations

Our study findings indicate that doctors are initiating patients with more advanced disease than nurses, which is consistent with findings from some previous studies [4]. This suggests that nurses initiate patients with earlier stage disease, allowing doctors to concentrate their efforts on those with more advanced disease or more serious illness. This may affect the down referral of patients from doctors to nurses and may also impact the interpretation of patient outcomes when comparing nurse and doctor ART initiations.

### Nurse Confidence

The NIMART mentorship program aimed to improve nurse confidence in HIV-related services for children, pregnant women, blood result interpretation and long-term side effects, suggesting a successful design of the training curriculum as these areas all showed significant improvements in nurse confidence level for the post-survey. It is important to note that these areas of greatest improvement in nurse confidence had the lowest confidence scores before training.

It is noted that some areas that did not see a significant increase in confidence level, such as TB screening and Cotrimoxazole prescription in adults, are in line with our findings in terms of areas needing improvement. The mean score for nurse confidence level in these areas was high in the pre-test (3.5, 3.4, respectively), possibly contributing to the lack of significant increase post-training. Certain areas, such as calculation of creatinine clearance (CrCl), had a highly significant improvement in nurse confidence level; however, that did not appear to translate into action. These factors will need to be taken into consideration for future mentorship trainings, being aware that confidence is not necessarily equal to competence and completion of routine practices.

Many clinical tasks related to paediatric HIV and treatment had low confidence scores on the pre-test and substantial improvement in the post-test; however, we did not specifically evaluate whether this resulted in more paediatric ART initiations. This is important as paediatric ART management has proven to be an especially challenging area in resource-limited settings, and the literature on task shifting of ART management for children is limited [15], [26]. The implications for children's access to HIV treatment suggest that nurses in the study are now more confident, and potentially more competent, in handling paediatric HIV patients, improving quality of patient care and treatment. These results are a testament to the valuable role that mentorship can play in improving access to paediatric ART.

This research provides insightful feedback on the performance of NIMART graduates in the clinical management of ART and the quality of care provided. Generally the nurses report feeling empowered by the additional knowledge [27]. With the lack of a doctor's daily presence for many of the City of Cape Town clinics, this programme allows the nurse to manage stable patients without having to wait to discuss with a doctor. Doctor interactions are reserved for more complex patients, and most nurses seem happy with this distinction. The study was not designed as a comparison of quality of care between nurse and doctor; it did not investigate patient outcomes, nor did we look at the time to treatment initiation, all of which are important elements to consider when assessing appropriateness of task shifting to nurses for HIV care.

### Limitations

The results of this study are limited by the fact that data were collected retrospectively from patient folders. The data may therefore be inaccurate if certain tasks were completed but not documented. The results therefore also rely on the clinical stationery in use at the time, as specific forms were not created for this study. Slight differences in the ART stationery existed between patient folders. Specifically, the TB symptom list for some folders included a checklist of boxes to aid completion of the register, which may have influenced documentation of TB symptoms. Not all study clinics had the same level of doctor involvement, potentially influencing the ability of nurses to refer sicker patients, and all study conclusions are based on one nurse mentor. The small number of nurses reviewed (8 of 21) may limit generalizability of results.

This study was restricted to adult ART initiations, and paediatric ART initiations were not included in the folder review. Western Cape NIMART nurses were not authorized to initiate ART on paediatric cases at the time of the study. The Western Cape NIMART guideline changed in 2014 to allow NIMART nurses to initiate paediatric ART following further mentorship and assessment.

While this study was not designed to assess whether this programme is reproducible in other settings, this model can be adapted as needed to suit any context. The basic HIV course is simply a training on the basics of HIV, and there is flexibility in how this is achieved, so long as the course covers the basics of what HIV is, modes of transmission, diagnosis and treatment. For the Western Cape model, the nurse does not have to have her PALSA Plus training completed before the 40 hours of mentorship starts and NIMART mentorship sessions can be used in place of some PALSA Plus sessions. Both the HIV course and PALSA Plus content could take place during the one-on-one mentorship for contexts were a specific HIV course or PALSA Plus guidelines were not available.

### Conclusions

Implementation of a nurse-mentor driven NIMART mentorship programme led to competent nurse initiation of the majority of patients, enabling doctors to manage complex patient cases. Nurses improved their confidence in performing HIV related clinical tasks, nurses were initiating patients after mentorship and the quality of initiation and management was satisfactory. These results suggest that in regions where the HIV burden places large demands on health services, a nurse mentoring model could assist in ensuring system efficiency for task shifting.

Mentorship provides motivation for increased clinical confidence and improves the quality of clinical care provided by nurses in some areas, creating an opportunity for professional development through the provision of effective technical backup. Clinical mentorship should be considered an essential component of the public health approach to universal ART access in resource-limited settings. Further research is needed to determine whether NIMART mentorship for nurses improves linkage to and retention in ART care.

## Acknowledgments

The authors would like to acknowledge research assistants, **Nikiwe Mkhosana** and **Nombulelo Raphahlelo**, of Médecins sans Frontières, Khayelitsha, as well as the dedicated staff at **Khayelitsha City of Cape Town clinics** who were involved in this study.
